# Development of Coarse-Grained Lipid Force Fields Based on a Graph Neural Network

**DOI:** 10.1021/acs.jctc.5c01071

**Published:** 2025-09-10

**Authors:** Zhenyu Liao, Ting Si, Tairan Wang, Ji-Jung Kai, Christophe Chipot, Jun Fan

**Affiliations:** † Department of Materials Science and Engineering, 53025 City University of Hong Kong, Kowloon 999077, Hong Kong China; ‡ Department of Physics, City University of Hong Kong, Kowloon 999077, Hong Kong China; § Department of Mechanical Engineering, City University of Hong Kong, Kowloon 999077, Hong Kong China; ∥ Centre for Advanced Nuclear Safety and Sustainable Development, City University of Hong Kong, Kowloon 999077, Hong Kong China; ⊥ Laboratoire International Associé Centre National de la Recherche Scientifique et University of Illinois at Urbana−Champaign, Unité Mixte de Recherche 7019, Université de Lorraine, BP 70239, F-54506 Lorraine, France; ■ Department of Biochemistry and Molecular Biology, University of Chicago, Chicago, Illinois 60637, United States; ▲ Theoretical and Computational Biophysics Group, Beckman Institute, and Department of Physics, University of Illinois at Urbana−Champaign, Urbana, Illinois 61801, United States

## Abstract

Coarse-grained (CG) lipid models enable efficient simulations of large-scale membrane events. However, achieving both speed and atomic-level accuracy remains challenging. Graph neural networks (GNNs) trained on all-atom (AA) simulations can serve as CG force fields, which have demonstrated success in CG simulations of proteins. Herein, we built data sets of AA simulations of DOPC, DOPS, and mixed DOPC/DOPS lipid bilayers and developed the first GNN-based CG lipid models based on the TorchMD-GN architecture. The CG lipid models reproduce the structural correlations of the AA simulations, accelerate the lipid dynamics by 9.4 times, and exhibit some degree of temperature transferability. Moreover, we demonstrate that training CG models on lipid bicelles enhances the performance of models in the lipid self-assembly and vesicle simulations. Our findings indicate that GNN-based CG lipid force fields show promise as a powerful approach for large-scale membrane simulations.

## Graphical abstract

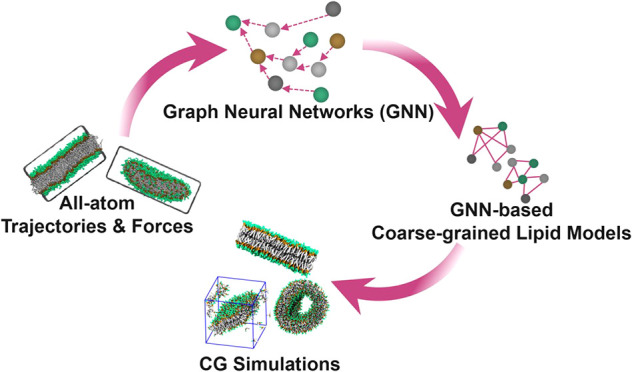

## Introduction

Cell membranes are essential for all living cells, serving not only as physical barriers but also as regulators in substrate transport and signal transduction.^1,2^ Understanding the mechanisms underlying the cellular processes on membranes is of great importance. Molecular dynamics (MD) simulations have emerged as a powerful tool for elucidating membrane-related processes at molecular resolution.^3–5^ All-atom (AA) simulations provide high-resolution details, but their computational cost becomes considerably high since many biological events on cell membranes take place over large spatiotemporal scales. To bridge this gap, coarse-grained (CG) force fields have been developed as an effective strategy in the membrane simulations.^6^

Given that lipids are the primary structural components of cell membranes, developing accurate and computationally efficient CG lipid force fields is of great importance. Based on parametrization strategies, CG force fields are mainly divided into two categories: top-down models and bottom-up models.^7^ The top-down models were parametrized to replicate macroscopic properties. A notable example is the Martini force field, which was fitted by reproducing experimental partition coefficients.^8,9^ Martini is recognized as the most widely used CG lipid model and has been developed to the latest version, Martini 3.^10^ Despite its great success, the Martini model still exhibits certain structural and thermodynamic inaccuracies, such as the inability to accurately depict the phase behavior of lipids and the inaccurate decomposition of entropy and enthalpy in the potential of mean force (PMF).^11–13^

The bottom-up models are parametrized by replicating the microscopic properties predicted by fine-grained simulations, typically at the atomic level.^14^ The main task in developing bottom-up models is to formulate an effective CG Hamiltonian. An ideal CG Hamiltonian should reproduce the many-body PMF from AA simulations.^15^ However, due to limitations in sampling and the basis set of CG interactions, it is impractical to achieve an exact match of the many-body PMF. Therefore, the key challenge is to design effective basis functions to approximate the ideal CG Hamiltonian. The CG basis functions are typically represented in terms of pair potentials, such as B-spline basis functions in the force-matching method.^15^ Several strategies have been proposed to enhance the accuracy of the CG energies, including the introduction of virtual particles,^16,17^ incorporating higher-order potential terms to account for many-body effects,^18^ and the inclusion of explicitly expressed electrostatic terms.^19,20^ Machine learning (ML) techniques provide another viable strategy where neural networks are employed to replace conventional pair potentials. The neural network is trained on data sets obtained from more expensive methods and serves as the energy function for CG simulations.^21^

Graph neural network (GNN) exhibits satisfactory performance in predicting atomic energies and forces.^22,23^ This network has developed the variational force-matching method into an ML framework for the development of CG force fields.^24–29^ TorchMD-GN^26^ is a mature GNN architecture that demonstrates outstanding performance in protein thermodynamics. The advantage of the network lies in its multiple interaction blocks, which can implicitly capture many-body effects by aggregating neighbor information, thereby preserving the thermodynamics more faithfully.^18^ Although membranes play a critical role in biological systems, there have not been any ML CG force fields specifically for lipids.

In this work, we trained solvent-free CG models for lipid membranes with various compositions based on TorchMD-GN. We examined two types of homogeneous membranes, including DOPC and DOPS, as well as a heterogeneous membrane composed of a mixture of DOPC and DOPS. We built data sets of AA simulations for these lipid membranes and then mapped the AA lipids to a CG representation. We trained several GNN-based CG lipid force fields and evaluated them through CG simulations starting from different initial configurations. Our results show that TorchMD-GN can be trained as fast and accurate CG lipid force fields with some transferability. We anticipate that this work will contribute to the application of ML methods in CG lipid models.

## Theory and Methods

### Coarse-Graining of Lipids

CG modeling consists of two steps: mapping from atoms to CG beads and parametrization of CG interactions. In this work, each lipid from AA simulations was mapped to a six-bead CG model, using center-of-mass mapping. The detailed mapping scheme can be found in Figure S1 and Table S1. The phosphate and choline/serine moieties were represented by the headgroup (HG) bead, while the glycerol and ester groups were defined as the middle-group (MG) bead. The first half and second half of the lipid tail were represented by T1 and T2 beads, respectively. Compared with the five-bead model,^30,31^ lipid tails in this model are represented by more beads. The strategy maintains computational efficiency while providing a more accurate depiction of the structural features of tails, with the expectation of better performance in lipid packing and lateral organization.^16,32^ The six-bead model has a resolution compatible with the previously developed GNN-based CG protein models.^26,33^ Our lipid model employs six beads to represent a lipid of 50–60 heavy atoms, whereas the protein model uses one bead for around 8.4 heavy atoms. Both the lipid and protein models represent 8–10 heavy atoms with a single CG bead, facilitating their combined use in future CG simulations of membrane proteins.

In the parametrization of lipid models, the variational force-matching method proves to be effective.^15,34^ It minimizes the mean squared error between the mapped AA forces, and the CG forces derived from the CG force field, to find the optimal CG force field

1 $$\chi^{2}\left[U_{\mathrm{CG}}\right]=\frac{1}{3N}\sum_{I=1}^{N}\left\langle {\left|\Xi_{I}\left(F\left(r_{n}\right)\right)+\nabla_{I}U_{\mathrm{CG}}\left(R_{N};\theta \right)\right|}^{2}\right\rangle$$

where Ξ _*I*_(*F*(**r**_*n*_)) is the AA forces projected onto the CG beads; Ξ_*I*_ is the force mapping operator; *F*(**r**_*n*_) is the instantaneous forces collected from all-atom simulations; CG forces (−∇_*I*_ *U*_CG_(**R**_*N*_;θ)) are derived from the CG force field; and **R**_*N*_ is the CG configuration mapped from the AA configuration **r**_*n*_.

In the classical bottom-up lipid force fields, spline basis functions are typically used to describe nonbonded interactions, and their coefficients are optimized according to eq 1.^16^ However, since spline potentials are typically pairwise models, many-body effects are neglected, which can affect the accurate simulation of lipid behavior.^35^ GNN can replace the previous pairwise models by using a graph structure to learn information from surrounding multilayer beads to predict CG energy and forces, thereby capturing many-body effects.^18,26^ Variational force-matching can be transformed into a machine learning approach, where eq 1 serves as the loss function, continuously optimizing the network parameters to develop the GNN-based force field.^24^

### Graph Neural Network Training

Our CG lipid models were trained based on the TorchMD-GN^26^ architecture in the TorchMD-Net 2.0 ^36^ package. TorchMD-GN is a graph convolutional network inspired by SchNet.^37^ The training procedure was described as follows. First, the training set had to be prepared. We collected coordinates and forces from AA simulations of lipids. The coordinates and forces were then mapped to the CG beads. Before feeding the data into the network, we extracted the prior term from the training data to avoid exploring physically meaningless regions. The prior term included the bonded term and the repulsive term. The bonded term was approximated by the harmonic potential

2 $$V_{\mathrm{bonded}}\left(r\right)=k{\left(r-r_{0}\right)}^{2}+V_{0}$$

where *k* is the spring constant, *r* is the pairwise distance, *r*_0_ is the equilibrium distance, and *V*_0_ is the base potential. The repulsive term was represented by a function inspired by the repulsive term of Lennard–Jones potentials

3 $$V_{\mathrm{repulsive}}\left(r_{\mathrm{ij}}\right)=4\epsilon {r_{\mathrm{ij}}}^{-6}+V_{0}$$

where *r*_*ij*_ is the pairwise distance between beads *i* and *j*, ϵ is the repulsive constant, and *V*_0_ is the base potential. The prior term was parametrized through Boltzmann inversion using the mapped AA trajectories. Prior forces were then derived from the prior term. We subtracted the prior forces from the AA forces to obtain the delta force, which is then used for GNN training.

The input for training TorchMD-GN is Cartesian coordinates **R**_*N*_ and types of CG beads **Z**_*N*_. First, the input data was passed through an embedding layer to generate a molecular graph. Each node in the graph corresponded to a CG bead and was assigned an embedding feature vector. The edges of the graph are the pairwise distances between beads within the cutoff range. Then, the edge information is used by the interaction block to update the node features. The interaction block typically consists of multiple layers (four layers in this work), which gradually integrate information from distant bead pairs. Lastly, a multilayer perceptron contracted the node features into an energy scalar ***E***_CG_(**R**_*N*_). The CG forces **F⃗**_CG_(**R**_*N*_) are derived by calculating the gradient of the energy scalar with respect to the initial coordinates, which act as the output of the model. CG forces will also be used to compute the force-matching loss, thereby continuously optimizing the GNN.

All training was carried out using PyTorch^38^ version 2.3 in Python 3.12. Most of the hyperparameters are consistent with the original literature.^26^ For several key hyperparameters, we conducted a hyperparameter search. Since the test loss does not show much difference between successful and failed models, the hyperparameter search was based on the CG simulations produced by the trained models.^26,29,39^ The metrics used for hyperparameter optimization are the stability of the bilayer in the CG simulations and the ability to reproduce the static properties seen in all-atom simulations. The set of hyperparameters in Table S2 ensures stable CG simulations and closely matches the results of AA simulations. The training input file with the complete set of hyperparameters is available in Zenodo.

The GNN-based force field for each lipid membrane was trained two to four times with different random seeds. Quick evaluations using CG simulations revealed little difference among models with different seeds. One of the models was selected for follow-up validation simulations. The training curves for the selected models are shown in Figure S2. Each model was trained on two Nvidia GeForce RTX 2080 Ti GPUs, taking about 7 min per epoch.

### All-Atom Simulations

CG models were built for DOPC, DOPS, and a mixture of 1:1 DOPC/DOPS lipids. Corresponding all-atom (AA) simulations were prepared as training sets for each lipid model individually (Table 1). The initial configurations of lipid bilayers were generated using CHARMM-GUI Membrane Builder.^40^ Each bilayer system consists of 512 lipids arranged in a bilayer within a periodic box of 14 × 14 × 10 nm^3^. The membrane was solvated with TIP3P^41^ water molecules in a periodic box and then neutralized by adding 150 mM of sodium and chloride ions. Lipid bicelles were prepared from the equilibrated bilayers. The lipid bilayer was placed in a larger water box (18 × 18 × 10 nm^3^) to eliminate the periodic image effects. The bilayer then transformed into a bicelle during hundreds of nanoseconds of simulation.

**Table 1:** Training and Validation Simulations Related to Each GNN Model in This Work

Force Field	System	Beads	Replicas	Time (ns)
GN_DOPC^a^				
CHARMM36	Training Set	132184	2	500
GN_DOPC^a^	Bilayer	3072	10	2
GN_DOPC	Bilayer	3072	5	100
GN_DOPC	Self-assembly	3072	5	10
GN_DOPC	Vesicle	5346	4	4
GN_DOPC	Bilayer-to-vesicle	3072	5	2
CHARMM36	Temp. 270 K	132184	1	300
GN_DOPC	Temp. 270 K	3072	5	2
CHARMM36	Temp. 330 K	132184	1	300
GN_DOPC	Temp. 330 K	3072	5	2
Martini 3	Bilayer	14356	2	400
Martini 3	Self-assembly	11008	2	400
GN_DOPS				
CHARMM36	Training Set	140182	1	500
GN_DOPS	Bilayer	3072	5	2
GN_PCPS^b^				
CHARMM36	Training Set	140622	1	500
GN_PCPS	Bilayer	3072	5	2
GN_DOPC_BC^c^				
CHARMM36	Training Set	226182	1	500
GN_DOPC_BC	Self-assembly	3072	5	20
GN_DOPC_BC	Vesicle	5346	2	10
GN_DOPC_BC	Bilayer-to-vesicle	3072	5	10
GN_DOPS_BC				
CHARMM36	Training Set	232266	1	500
GN_DOPC_BC	Self-assembly	3072	3	10
GN_PSPC_BC				
CHARMM36	Training Set	231512	1	500
GN_DOPC_BC	Self-assembly	3072	3	10

a GN_* refers to the GNN-based CG models corresponding to lipids. All of the models are available in Zenodo.

b *_PCPS represents a 1:1 mixture of the DOPC/DOPS lipid membrane.

c *_BC means that the model was trained on bicelles.

All the simulations were carried out by GROMACS-2020 ^42^ along with the CHARMM36 force field.^43,44^ Energy minimization was performed on each system with 1000 KJ·mol^–1^·nm^–2^ harmonic restraints on the phosphorus atoms of lipids. The restrained system was heated to 300 K under *NPT* conditions for 2.5 ns. The restraints were then gradually relaxed, and two equilibrium simulations lasting 2.5 ns each were performed. Afterward, all restraints on the pre-equilibrated membrane were removed, and *NPT* simulations were continued until the membrane area reached equilibrium. Each membrane underwent a 300 ns *NPT* simulation. Lastly, a 200 ns *NVT* simulation was run to collect trajectories for CG force field development, storing the coordinates and forces every 0.02 ns. As a result, the database for each model contained 10,000 frames of coordinates and forces. Pressure is kept at 1 bar with a semi-isotropic Parrinello–Rahman barostat,^45^ while temperature is controlled by a Nose–Hoover thermostat.^46,47^ A smooth cutoff of 10–12 Å and the particle-mesh Ewald^48^ method were applied to treat van der Waals (vdW) and long-range electrostatic interactions, respectively. A time step was set to 2 fs, with the LINCS^49^ algorithm applied to constrain all covalent bonds involving hydrogen.

### Coarse-Grained Simulations

The details of the simulations are summarized in Table 1. In the bilayer simulation, the initial membrane configurations are consistent with the reference AA simulations, and the box dimensions correspond to those used in the AA simulations. In the lipid-assembly simulation, a number of lipids, equivalent to that in the training set, are randomly placed in an isotropic periodic box, with the side length matching that of the AA membrane plane. The vesicle is modeled using CHARMM-GUI and placed in a periodic box of 24 × 24 × 24 nm^3^. The bilayer-to-vesicle simulations start from the lipid bilayer in the training set placed in a large box (24 × 24 × 18 nm^3^). All the systems were modeled without the solvent.

CG simulations were conducted using TorchMD.^50^ The force field consists of two components: the force field trained by TorchMD-GN and the prior term. Each system underwent Langevin simulations under *NVT* conditions at the same temperature of 300 K as the AA simulations (unless otherwise noted). The damping constant is set to 0.1 ps^–1^. A time step is typically set to 1 or 20 fs.

The CG simulations using the Martini 3 force field followed the simulation protocol outlined in a previous benchmarking study of Martini 3.^11^ Each lipid model used in Martini 3 was mapped into the six-site model for data analysis, with mapping rules provided in Table S1.

### Analysis Details

All snapshots were visualized and rendered in VMD^51^ version 1.9.3. The radial distribution function is calculated by using the measure module of VMD. *Z*-density was defined as the distribution of the *z*-coordinates of the corresponding beads. All densities in this work have been area-normalized. The membrane thickness refers to the bin-averaged normal distance between the upper and lower HG beads, with each grid being 10 × 10 Å. Orientational order parameters were obtained from the second-order Legendre polynomial for cos­(θ) as follows

4 $$S\left(\theta \right)=\left\langle \frac{1}{2}\left(3\cos^{2}\left(\theta \right)-1\right)\right\rangle$$

where θ is the angle between the lipid normal and the vector of the T1–T2 (or HG-MG) bond. The range of order parameters is from −0.5 to 1, where the value of 1 indicates that the bond is parallel to the normal direction of the membrane, while the value of −0.5 indicates an orthogonal orientation. The bending modulus was calculated by the real-space fluctuation method.^52,53^ The lateral mean square displacement (MSD) was calculated by the following equation

5 $$\mathrm{MSD}\left(t\right)=\left\langle {\left(r_{i,\mathrm{xy}}\left(t+\tau \right)-r_{i,\mathrm{xy}}\left(\tau \right)\right)}^{2}\right\rangle$$

where *r*_*i,xy*_ is the *xy* coordinates of the MG beads for each lipid, τ is the initial time point, and *t* is the time interval. The diffusion coefficient was defined as a quarter of the slope of the lateral MSD curve. The order parameters and lateral MSD were both averaged over all lipids in the trajectories after reaching equilibrium.

## Results

The workflow for developing and evaluating GNN-based CG lipid force fields is illustrated in Figure 1. First, AA simulations of lipid bilayers were conducted, and the coordinates and forces were recorded. These coordinates and forces were then mapped to the CG representation. The mapped coordinates and their associated CG bead types were fed into a GNN architecture, TorchMD-GN. The input data went through an embedding layer, multiple interaction blocks, and an output layer and then produced the energy and force for each bead. The model was trained by minimizing the mean squared error loss between the delta forces and the predicted forces from the model, where delta forces refer to the mapped forces obtained after subtracting the prior energy terms. Lastly, extensive CG simulations were performed to systematically evaluate the capabilities and limitations of the GNN-based GG lipid models. More details about the workflow can be found in the Theory and Methods section.

**Figure 1:**
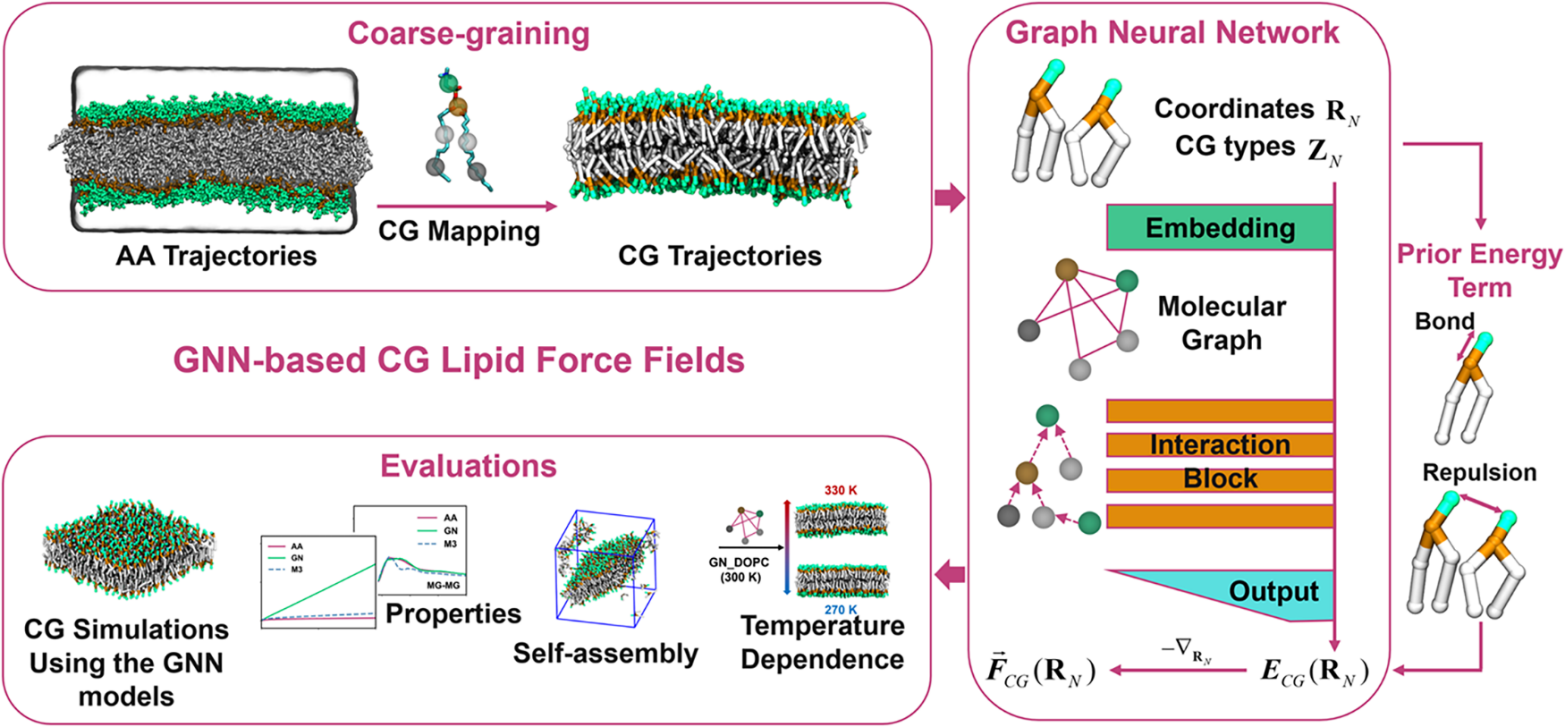
Flowchart of developing and testing GNN-based CG lipid force fields.

### Structural and Dynamic Properties of Lipids in the GN_DOPC Model

DOPC is a very common phospholipid in cell membranes and has been widely used as a template in the development of the CG force field.^16,17,54^ Therefore, we first trained a GNN-based CG force field for DOPC lipids, referred to as GN_DOPC. To evaluate the GN_DOPC model, we conducted ten replicas of 2 ns CG simulations (with a time step of 1 fs) and five replicas of 100 ns simulations (with a time step of 20 fs). We performed a systematic analysis of the simulation results, comparing them with the all-atom data from the training set and the outcomes of CG simulations with Martini 3.

First, the stability of the GN_DOPC model was tested. The snapshot (Figure 2a) and movie of the simulation trajectories (Video S1) show that the bilayer structure remains stable throughout the simulations. We conducted a time-dependent analysis. Figure S3 illustrates that the potential and total energies rapidly stabilize during all of the simulations. In addition, we also plotted the time evolution of the membrane properties, which showed no significant changes over time (Figure S4a–c). The membrane is also stable in the CG simulations with a longer time step (Figure S4d,e). While two of the lipids drift into the vacuum, the majority of the lipids maintain the bilayer structure.

**Figure 2:**
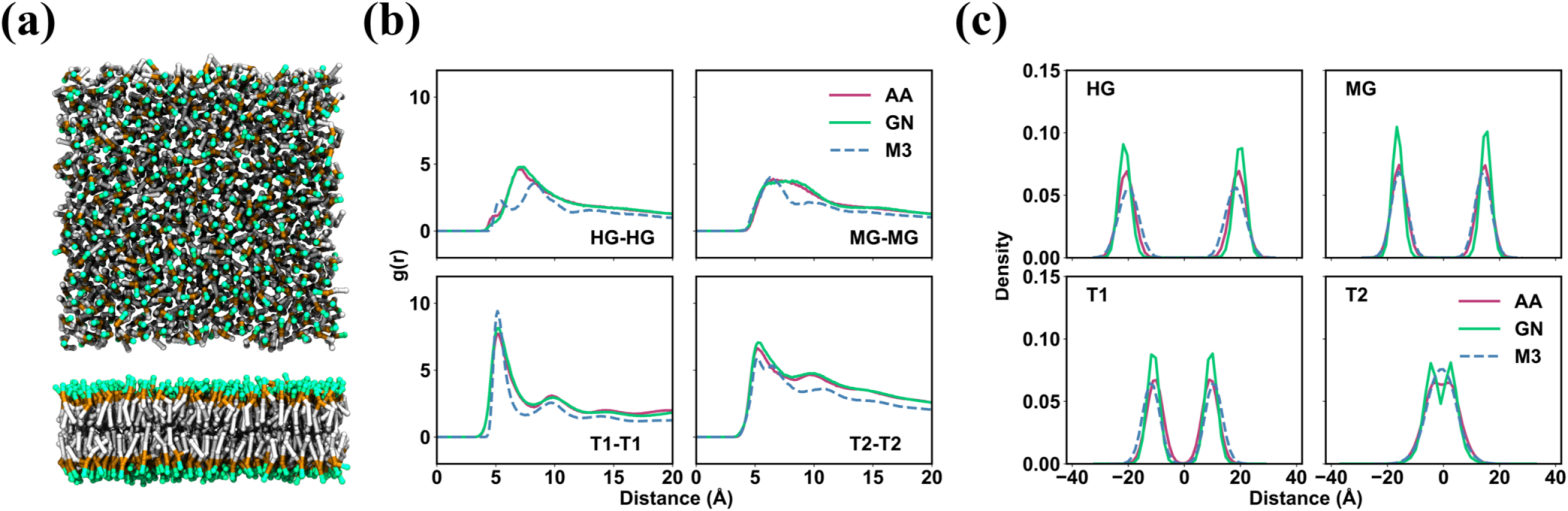
Structural features of the GN_DOPC model. (a) Snapshots of the equilibrated DOPC lipid bilayer from CG simulations using the GN_DOPC model. (b) RDFs between the same type of beads and (c) *z*-density distribution of beads from the mapped AA simulations, the CG simulations using the GN_DOPC model, and the CG simulations with Martini 3.

Second, the structural correlation of the GN_DOPC model between CG and AA simulations was examined. The distributions of bonds and angles in the CG simulations are consistent with those in the AA model, except for the T1–T2 bonds (Figure S5a,b). The inset in Figure S5a shows that due to the flexibility of the lipid tails, an additional peak in the distribution of the T1–T2 bonds from the mapped AA trajectories appears around 4.5 Å. This peak is difficult to capture due to the harmonic potential of the prior bonded term. To address the issue, we attempted to remove the prior term and trained a network directly from the mapped AA forces. The results of the simulations show that the absence of the prior term leads to significant distortion of the membrane, with some bonds becoming overly stretched (Figure S6). We also investigated B-spline potentials for describing the bonded term. For the T1–T2 bond, the B-spline potential exhibits a local minimum at 4.4 Å, which is expected to better capture the non-Gaussian distribution of the T1–T2 bonds (Figure S5c). These results indicate that it is still necessary to describe the bonded term with a basis set when training CG lipid force fields, but a more flexible functional form, such as B-splines, is recommended to explore.

The nonbonded structural correlation was assessed by radial distribution functions (RDFs). The RDFs for beads in both AA and CG (using GN_DOPC) simulations exhibit a high degree of consistency, which demonstrates that GN_DOPC can reproduce pair correlations in the AA model (Figures 2b and S7). In contrast, the RDFs for beads in the CG simulations with Martini 3 are notably different from those in the AA simulations.

The membrane structure from the GN_DOPC model was also characterized. We observed that the *z*-density distributions under CG become narrower and higher compared to those under AA, with the peak position of each bead moving outward from the center of the membrane by 1 Å (Figure 2c). The *z*-density profile of the T2 beads shows a separation of 1 Å between the upper and lower lipid layers, a feature that is absent in the AA model. Other structural properties are presented in Table 2. The thickness (*d*_HG–HG_), tail order parameter (*S*_TT_), and bending modulus (κ_Bend_) in the GN_DOPC model are slightly greater than those in the AA model, suggesting that the membrane becomes somewhat thicker and more ordered. The headgroup order parameter (*S*_HM_) in the GN_DOPC model shows good agreement with the AA model, indicating that the GN_DOPC model effectively captures the conformation of the head groups. There are also differences in membrane structural properties between the Martini and AA models, such as the peak positions of bead *z*-density shifting inward by 1 Å, reduced membrane thickness, and lower headgroup ordering. The calculation of the membrane structural properties indicates that there is still room for improvement in the fitting of the bead distribution along the normal direction of the DOPC lipid bilayer.

**Table 2:** Properties of Lipid Bilayers, Including Bilayer Thickness (*d*_HG–HG_), T1–T2 (*S*_TT_) and HG-MG (*S*_HM_) Order Parameters, Bending Modulus (κ_Bend_), and Diffusion Constant (*D*_C_) from Experiments and Computational Models

Status	*d*_HG–HG_ (Å)	*S*_TT_	*S*_HM_	κ_Bend_ (*k*_*B*_ *T*)	*D*_C_ (10^–7^ nm/s)
Expt. (DOPC)	44.8^a^	N.A.	N.A.	18.3^a^	0.82^b^
AA (DOPC)	40.2	0.44	0.57	18.8 ± 0.1	0.86 ± 0.02
GN_DOPC	41.2	0.60	0.55	27.8 ± 0.3	78.53 ± 0.47
Martini (DOPC)	38.6	0.43	0.26	15.5 ± 0.1	5.99 ± 0.05
AA (DOPS)	43.5	0.54	0.68	21.7 ± 0.1	0.21 ± 0.02
GN_DOPS	43.0	0.58	0.64	29.6 ± 0.1	44.64 ± 2.35
AA (PC/PS)	42.3	0.50	0.68	17.6 ± 0.4	0.29 ± 0.01^c^
CG_PCPS	42.8	0.60	0.65	29.9 ± 0.3	42.92 ± 2.75^d^

a Ref 55.

b Ref 56.

c *D*_C_ (PC) = 0.30 ± 0.01 × 10^–7^ nm/s; *D*_C_ (PS) = 0.27 ± 0.01^–7^ × 10^–7^ nm/s.

d *D*_C_ (PC) = 42.82 ± 2.01 × 10^–7^ nm/s; *D*_C_ (PS) = 43.03 ± 2.05^–7^ × 10^–7^ nm/s.

Third, the dynamic properties of lipids are evaluated by the lateral mean square displacement (MSD) plot (Figures 3 and S8) and the diffusion constant (*D*_C_) (Table 2). The lateral diffusion of lipids in the AA model is relatively slow with a diffusion constant of 0.86 ± 0.02 × 10^–7^ nm/s. The diffusion constant from AA is consistent with the experimental results^56^ and another simulation study^57^ using the CHARMM36 force field. The lipid diffusion is accelerated in the Martini 3 model, with a diffusion coefficient that is 8 times that of AA. The lipid diffusion in the GN_DOPC model is roughly 100 times faster than in the AA model due to its highest degree of coarse-graining.

**Figure 3:**
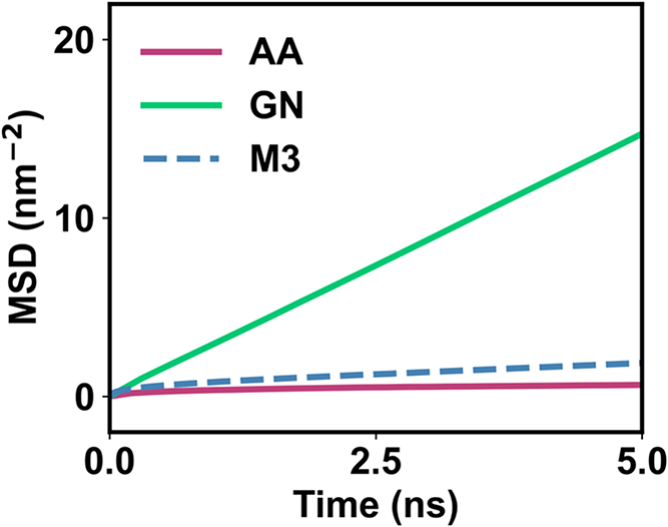
Dynamic properties of the membrane, represented by the lateral mean square displacement (MSD) of lipids obtained from the AA simulations, the CG simulations using the GN_DOPC model, and the CG simulation with the Martini 3 model.

We evaluated the efficiency of the GN_DOPC model and the AA model using the MSD values and wall-clock times (Table S3). The wall-clock time of the GN_DOPC model is similar to that of the AA model. However, the diffusion of lipids in the CG model is much faster, exhibiting a speed-up of 9.4 times.

### Extension to Charged and Mixed Lipids

Developing CG force fields for charged lipids is nontrivial and often requires special treatment of electrostatic interactions.^19^ Since membranes typically consist of various lipid types, it is essential to accurately describe the interactions between these different lipids. Therefore, we used the process of developing the GN_DOPC model to build CG force fields for charged and mixed lipids. DOPS is an anionic lipid with a charged head, which plays a vital role in cellular processes such as viral invasion and protein recognition.^58,59^ Thus, we developed a GN_DOPS model for the DOPS lipid bilayer and a GN_PCPS model for the mixed 1:1 DOPC/DOPS membrane. Snapshots and movies reveal that the GN_DOPS and GN_PCPS models can still produce lipid bilayer structures, but their stability is not as good as that of the GN_DOPC model (Figure 4a,b and Videos S2 and S3). In particular, these models show defects such as lipid heads entering the hydrophobic zone, tails protruding into the vacuum, and lipids detaching from the membrane. Time-dependent analysis shows that the membrane is stable overall, without significant changes in properties over time (Figure S9).

**Figure 4:**
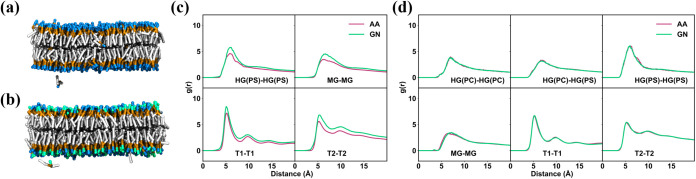
Extension to charged and mixed lipids. Snapshots of the (a) DOPS and (b) 1:1 DOPC/DOPS lipid bilayer from the simulations using the GN_DOPS and GN_PCPS models, respectively. RDFs between selected beads for the (c) DOPS and (d) DOPC/DOPS bilayer from the mapped AA simulations and the CG simulations using the GNN-based lipid models.

We analyzed the structural and dynamic properties of the lipids from the GN_DOPS and GN_PCPS models. The RDF analysis indicates that the GN_DOPS model captures the peak distances but overestimates their magnitudes (Figures 4c and S10a). In contrast, the GN_PCPS model perfectly matches the distribution of beads from the AA results (Figures 4d and S10b). The *z*-density distributions reveal that the results from the GN_DOPS and GN_PCPS models are largely consistent with the AA results, although some overly structured features seen in the GN_DOPC model are also present (Figure 5). The thickness of the DOPS and DOPC/DOPS membranes, as well as the ordering of the head groups, remains consistent between CG and AA (Table 2). However, the ordering of the tails and the bending modulus suggest that the membranes become slightly stiffer. Similar to the GN_DOPC model, lipids in these two models exhibit lateral diffusion much faster than that in the AA simulations.

**Figure 5:**
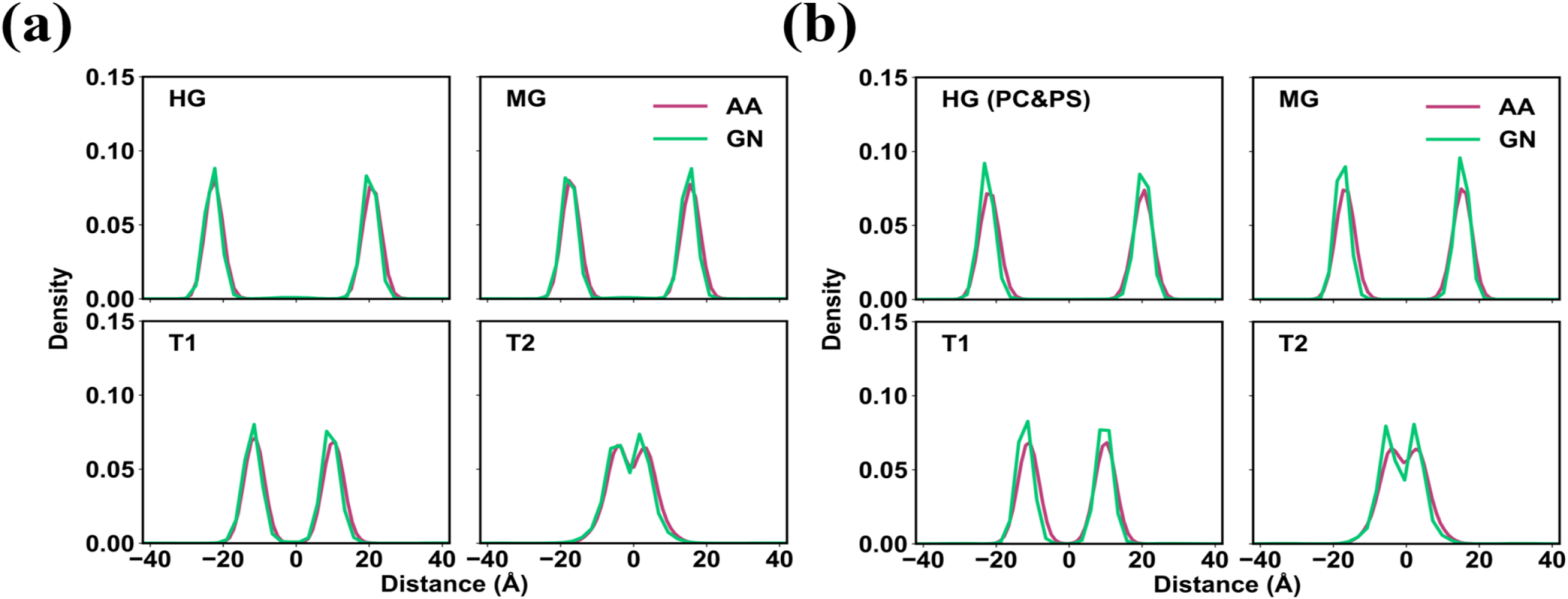
*Z*-density profiles for the (a) DOPS and (b) DOPC/DOPS lipid bilayer from the mapped AA simulations and the CG simulations using the GNN-based lipid models.

### Training on Lipid Bicelles Improves the Lipid Self-Assembly Behavior

The results above suggest that the GNN-based CG lipid models trained according to our proposed process show satisfactory performance on the training set, efficiently capturing certain structural correlations of AA. It is unclear whether the models can apply to systems outside the training set. Therefore, we performed self-assembly, vesicle, and bilayer-to-vesicle simulations with the GN_DOPC model. The initial configurations for these simulations can be found in Figure S11, and the system setup is detailed in the Theory and Methods section. The simulation results indicate that GN_DOPC does not fully capture the amphipathic behavior of the lipids (Figure 6). In the self-assembly simulation, the lipids form an incomplete bilayer structure. In the vesicle simulations, the vesicle struggles to maintain stability and ultimately disintegrates into unassembled lipids. When a bilayer is placed in a larger box to block its view of periodic images, the lipids do not form bicelles or vesicles; instead, they disassemble.

**Figure 6:**
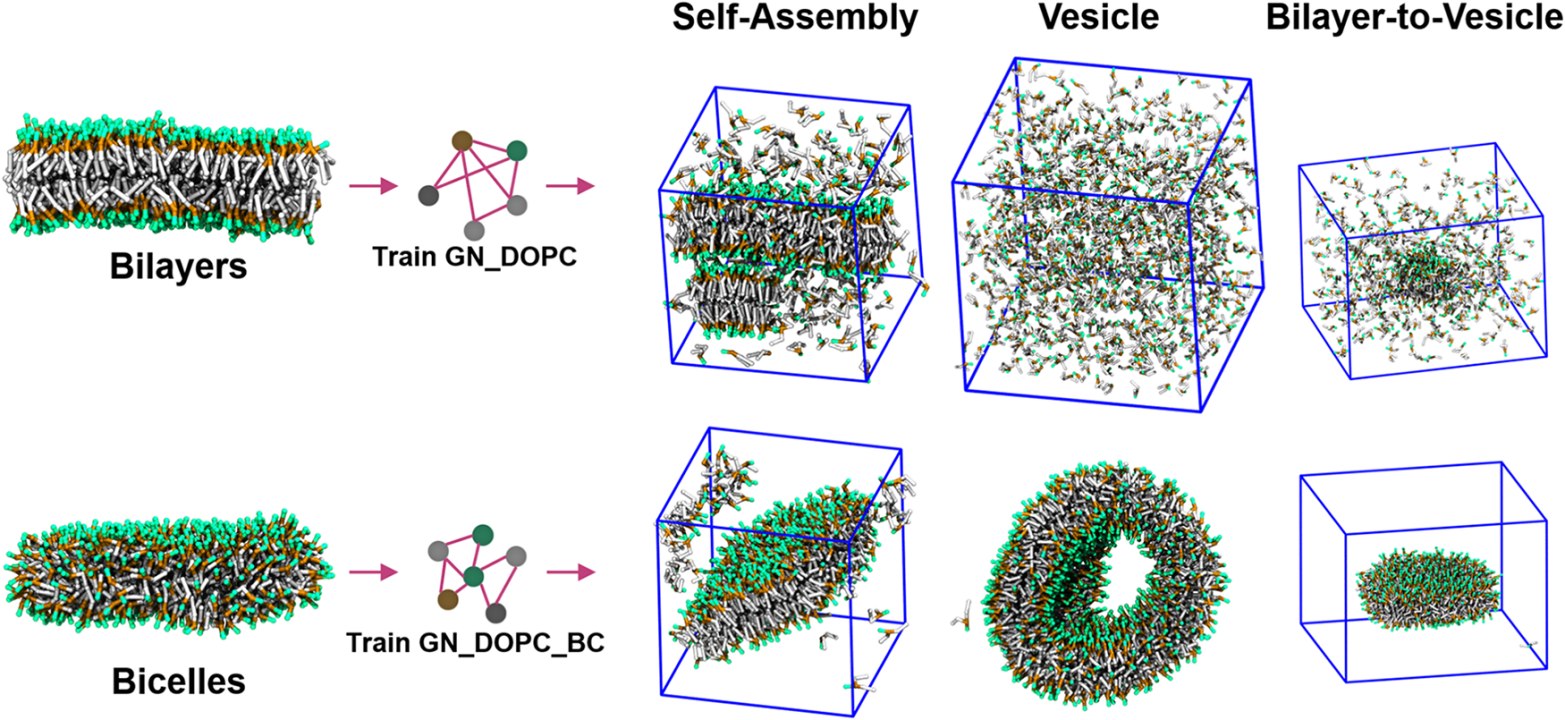
Training CG lipid models on lipid bicelles improves the performance of the force field in the self-assembly, vesicle, and bilayer-to-vesicle simulations. Snapshots of the outcome of each simulation were presented.

The bilayer-to-vesicle simulations offered insight: the GN_DOPC model has only learned the lipid configurations within the bilayer, and it cannot capture the curved region in the bicelles or vesicles. To enhance the generalization ability of the CG models, we established a new training set. We placed the lipid bilayer from the training set in a large water box to eliminate the periodic image effect. In the 400 ns AA simulations, the lipid bilayer transformed into a disc-like structure known as the lipid bicelle. We trained a new GNN-based CG lipid force field on the bicelles (GN_DOPC_BC) and applied it to the aforementioned simulations. The training curves for the new models can be seen in Figure S12. The GN_DOPC_BC model achieves the self-assembly of lipids and demonstrates its stability in vesicles and bicelles (Figure 6 and Videos S4, S5, and S6). In the self-assembly simulations, the GN_DOPC_BC model can better capture the pair correlations in AA simulations of the lipid bilayer compared to Martini (Figure S13).

We also trained a GN_DOPS_BC model with DOPS bicelles and a GN_PCPS_BC model with 1:1 DOPC/DOPS bicelles and tested their quality in self-assembly simulations (Figures S12 and S14). Movies of trajectories show that the free lipids quickly aggregate into a bilayer under these two models (Videos S7 and S8). These results suggest that training on lipid bicelles, which have richer lipid configurations, seems to be a more reliable method for developing GNN-based CG lipid force fields. The new trained models demonstrate a stronger ability to understand the amphipathic behavior of lipids.

### Transferability of Temperature

One of the applications of CG lipid force fields is to study lipid phase transition,^13,54,60^ so it is essential to explore whether our models can accurately capture lipid phase behavior. Experimental evidence indicates that DOPC lipids gradually become more ordered until a liquid-to-gel phase transition occurs at 257 K.^60–62^ Given that the phase transition occurs at a rather low temperature, we carried out several temperature-dependent simulations to probe the temperature transferability of our GNN-based CG lipid force fields within the liquid phase. The GN_DOPC model trained at 300 K was employed to simulate the lipid bilayer at 330 and 270 K (Figure 7a). The snapshot of simulations shows that in the CG simulation with GN_DOPC, the stable bilayer structure of the membrane is maintained at the higher and lower temperatures (Figure 7a). The calculation of membrane properties indicates that both AA and CG simulations show very similar trends: as the temperature decreases, lipid diffusion slows down, and the membrane becomes thicker and more ordered (Figure 7b–d).

**Figure 7:**
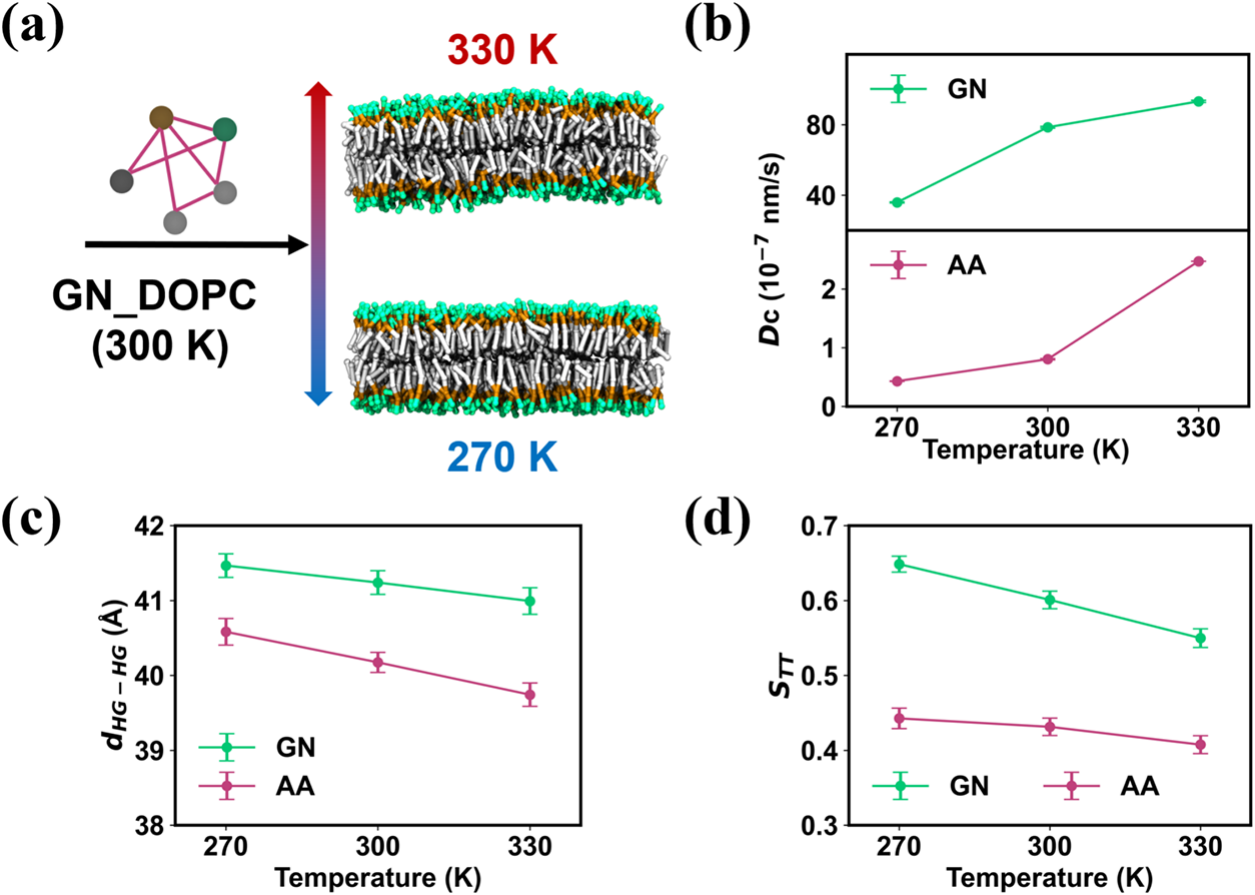
Transferability performance of the GN_DOPC model under different temperatures. (a) Snapshot derived from CG simulations of the DOPC lipid bilayer at 330 and 270 K, with the GN_DOPC model trained from trajectories obtained from AA simulations at 300 K. The properties of the membrane, such as (b) diffusion constant, (c) membrane thickness, and (d) order parameter, are compared between CG with GN_DOPC and AA simulations at 270, 300, and 330 K.

## Discussion and Conclusions

In light of the impressive success of TorchMD-GN in developing CG protein force fields, we applied this framework to the development of lipid force fields in this work. As the first endeavor to develop GNN-based CG lipid force fields, we have demonstrated the considerable promise of machine learning in the lipid system while also recognizing several issues that require improvement.

GNN-based CG lipid force fields demonstrate considerable accuracy in CG simulations of membranes. The CG simulations almost reproduce the distribution of bonds and angles as well as RDFs between beads in the AA simulations. The accuracy is comparable to that of traditional bottom-up CG models, showing the feasibility of using GNN as potential functions.^16,17,54^ However, our models slightly overestimate the membrane thickness and ordering of the lipid tails. Similar trends have also been reported in previous bottom-up models.^16,32,63^ For the GNN model, we suggest improving the limitation in two ways. The bonded term should be optimized by modeling with more flexible potential functions or being learned directly by GNN. On the other hand, the environment of the lipid tails is relatively complex, with neighbors that include particles from both the same layer and the opposite layer. Therefore, more training data may be required for the GNN to accurately learn the information on lipid tails.

GNN-based CG lipid force field also ensures computational efficiency. The wall-clock time of CG simulations with our models is similar to that of AA simulations since each step requires loading the network and calculating the forces. However, due to fewer degrees of freedom and a smoother free-energy landscape in CG simulations, each step corresponds to a longer physical time equivalent. When the same degree of lipid diffusion is achieved, our model is significantly faster than the AA force field. The accelerated lipid diffusion makes our models more suitable for large-scale, multicomponent complex membrane simulations. As TorchMD continues to improve,^50^ we anticipate that our model will become more efficient in the future.

Our training framework is also effective for charged and mixed lipids. The GN_PCPS model successfully reproduces the pair correlations from AA simulations and distinguishes the distributions of DOPS and DOPC lipids, which is evident in the different peak heights in the HG-related RDFs (Figure 4d). These findings show that TorchMD-GN can easily develop CG lipid models for lipid membranes of any composition, demonstrating the broad applicability of the framework.

We also found that training with bicelles improved the self-assembly and membrane-bending behaviors of lipids. The training set of bicelles features richer lipid configurations. 75% of the total lipids remain a planar bilayer, while 25% of them cover the curved edge of bicelles (Figure S15). In contrast, there are almost no such edge lipids in the bilayer training set. The results indicate that enriching the training set with varied lipid configurations can enhance the model performance. Future studies could consider incorporating more diverse membrane configurations, such as vesicles, into the training set.

The stability of the GN_DOPC_BC model in vesicles and bicelles makes it promising in large-scale membrane simulations, such as whole-cell,^5^ virion,^64^ and nuclear pore complex^65,66^ simulations. Before implementing this in large biological systems, it is essential to model the interactions of lipids with proteins. The next step is to develop a universal GNN-based CG force field for proteins and lipids. Our model also demonstrates a degree of temperature transferability attributed to the fitting of the PMF that incorporates an entropy term, which captures some temperature dependence. However, developing a truly temperature-transferable lipid force field is no easy task and requires the correct partitioning of enthalpic and entropic interactions.^67,68^ The applicability of this framework to lipids with complex phase behavior, such as DPPC, deserves an independent study.

## Supplementary Material



















## Data Availability

Input files for training and CG simulations, GNN-based CG lipid force fields, mapping script, and initial configuration files of simulations are available at 10.5281/zenodo.16792306

## Notes

Additional information on the CG mapping scheme, hyperparameter search, training curves, time-dependent analysis, bond and angle distributions, RDFs between different types of beads, MSD values of different groups of lipids, initial configurations of lipids, snapshots of lipid aggregation behavior, and edge lipid and planar lipid in the bicelle (PDF)
Trajectory of the DOPC lipid bilayer from simulation using GN_DOPC (Video S1) (AVI)
Trajectory of the DOPS lipid bilayer from simulation using GN_DOPS (Video S2) (AVI)
Trajectory of the DOPC/DOPS lipid bilayer from simulation using GN_PCPS (Video S3) (AVI)
Trajectory of DOPC lipids from self-assembly simulation using GN_DOPC_BC (Video S4) (AVI)
Trajectory of the DOPC lipid vesicle from simulation using GN_DOPC_BC (Video S5) (AVI)
Trajectory of DOPC lipids from bilayer-to-vesicle simulation using GN_DOPC_BC (Video S6) (AVI)
Trajectory of DOPS lipids from self-assembly simulation using GN_DOPS_BC (Video S7) (AVI)
Trajectory of DOPC/DOPS lipids from self-assembly simulation using GN_PCPS_BC (Video S8) (AVI)
