# Supplementary material for: Development of Coarse-Grained Lipid Force Fields Based on a Graph Neural Network
Source: J Chem Theory Comput. 2025 Sep 10;21(18):9175–85. doi: 10.1021/acs.jctc.5c01071 (PMC12487984; doi:10.1021/acs.jctc.5c01071)
Supplement: Supplementary file 1 [file ct5c01071_si_001.pdf]

Development of Coarse-Grained Lipid Force Fields Based on a Graph Neural Network

Zhenyu Liao[1], Ting Si [1,2], Tairan Wang[1], Ji-Jung Kai [3,4], Christophe Chipot[5], Jun Fan[1,3,4] ⋆

[1] Department of Materials Science and Engineering, City University of Hong Kong, Kowloon 999077, Hong Kong, China;

[2] Department of Physics, City University of Hong Kong, Kowloon 999077, Hong Kong, China;

[3] Department of Mechanical Engineering, City University of Hong Kong, Kowloon 999077, Hong Kong, China;

[4] Centre for Advanced Nuclear Safety and Sustainable Development, City University of Hong Kong, Kowloon 999077, Hong Kong, China;

[5] Laboratoire International Associé CNRS and University of Illinois at Urbana−Champaign, UMR no. 7019, Université de Lorraine, BP 70239, F-54506 Vandœuvre-lès-Nancy, France.

Corresponding Author

⋆ J. Fan: junfan@cityu.edu.hk

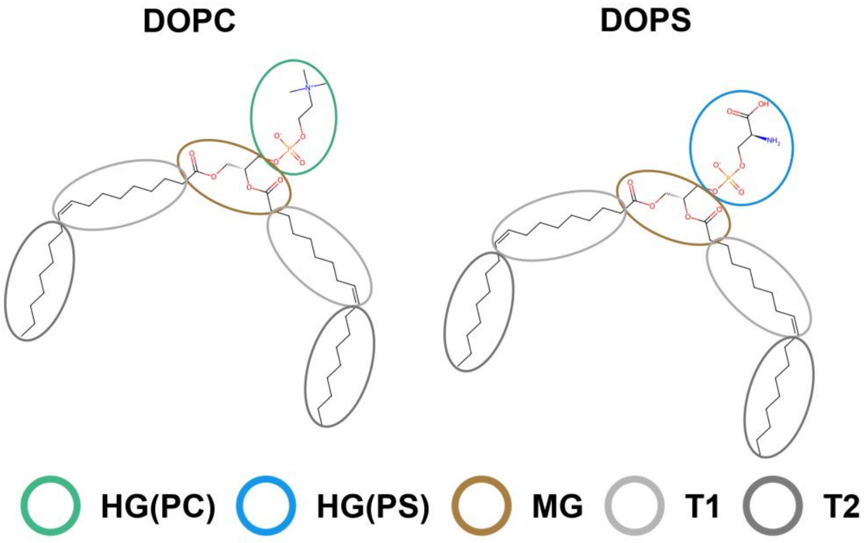

Figure S1. The mapping scheme for the (a) DOPC and (b) DOPS lipids.

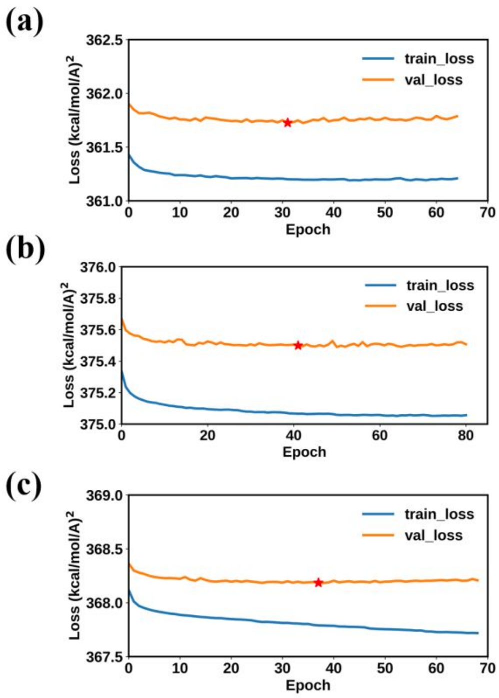

Figure S2. Training and validation loss as a function of the training epoch number for the (a) GN_DOPC, (b) GN_DOPS and (c) GN_PCPS model. The selected model is marked with a red star.

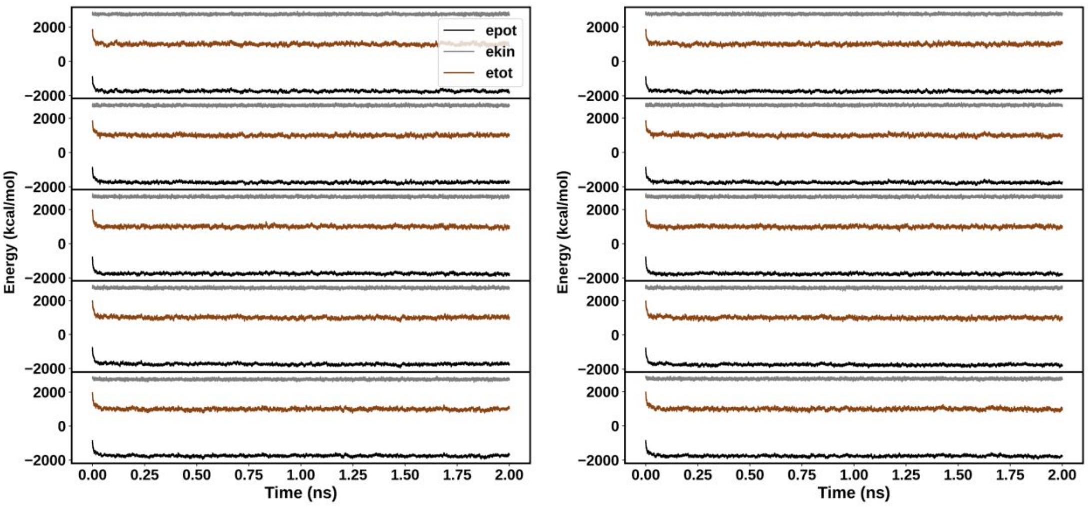

Figure S3. Time evolution of potential Energies (epot), kinetic energies (ekin) and the total energies (etot) throughout ten replicas CG simulations of DOPC lipids.

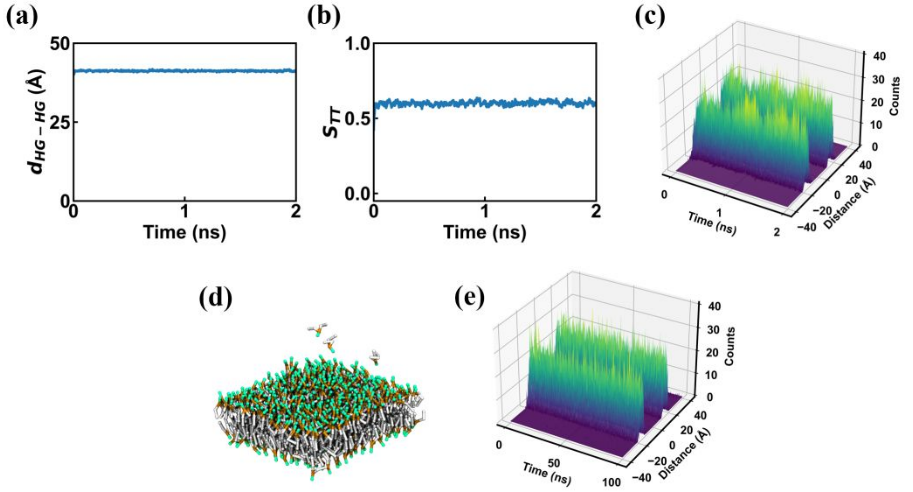

Figure S4. Time-dependent analysis of the GN_DOPC model. Time evolution of (a) thickness, (b) order parameter and (c) Z-density of MG beads, in the CG simulations with GN_DOPC. (d) A snapshot from the outcome of CG simulations with a 20-fs timestep, and the temporal changes in Z-density of MG beads in the simulation.

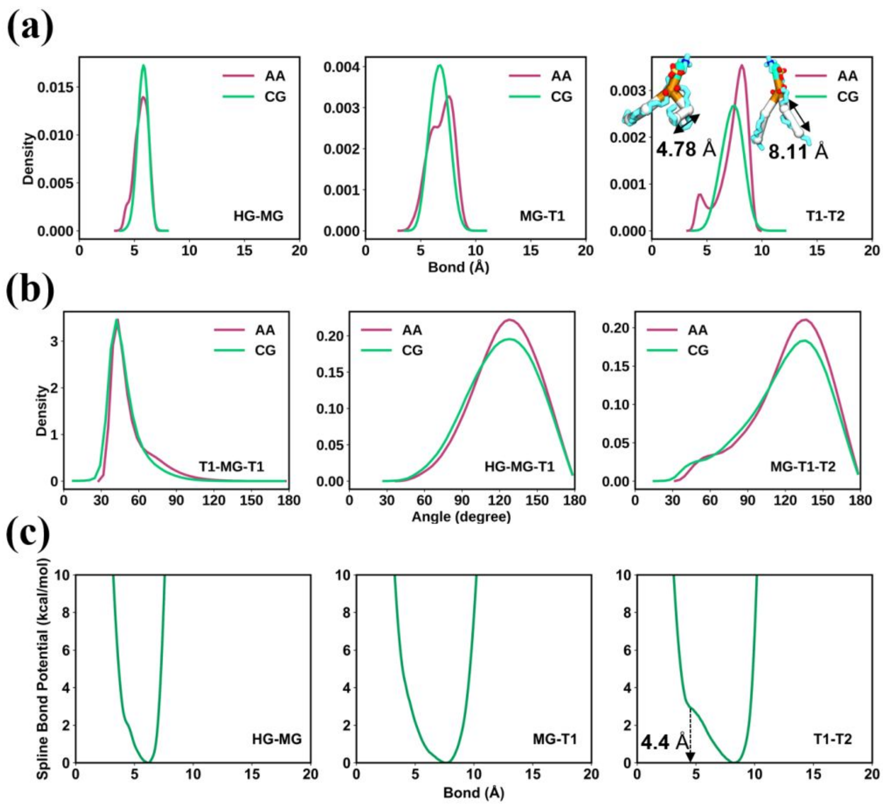

Figure S5. (a) Bond and (b) angle distributions of DOPC lipids from the mapped AA and CG simulations with GN_DOPC. The inset in (a) illustrates two distinct T1-T2 bonds in the mapped AA trajectories: the bond length is 4.78 Å for bent lipids, whereas it is 8.11 Å for non-bent lipids. (c) Third-order B-splines of the bonded terms.

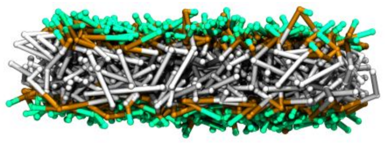

Figure S6. A snapshot of DOPC lipids from the CG simulations using the GNN model trained without the prior term.

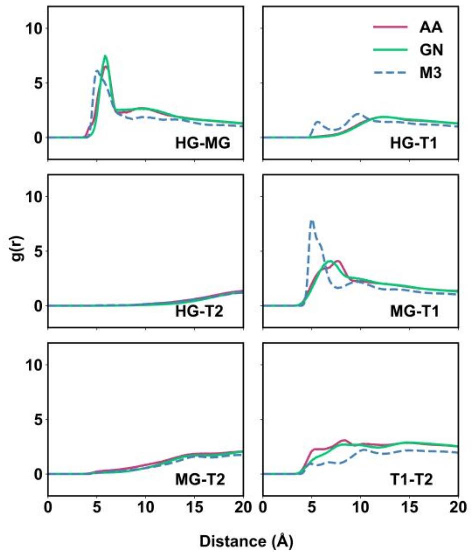

Figure S7. The RDFs between different types of beads from the mapped AA simulations, the CG simulations using the GN_DOPC model, and the CG simulations with Martini 3.

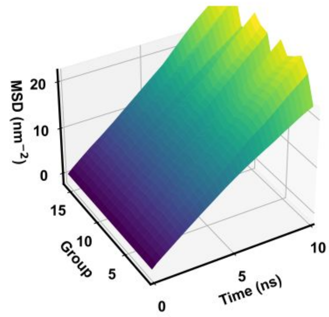

Figure S8. The MSD values of different groups of lipids in the CG simulations with GN_DOPC. The 512 lipids were divided into 16 groups, and the MSD value for each group was calculated. The MSD values between different lipid groups are very similar, demonstrating the reasonability of calculating the MSD value using all the lipids in Figure 3 in the main text.

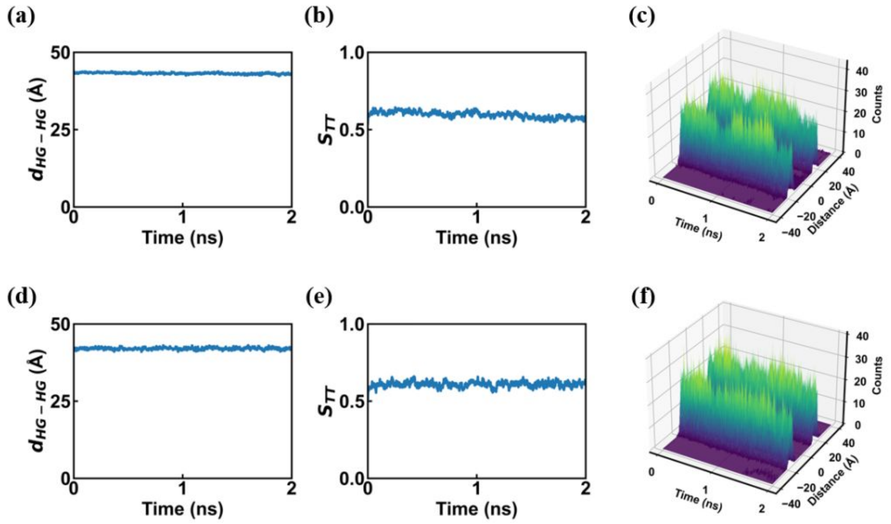

Figure S9. Time-dependent analysis. Time evolution of (a) thickness, (b) order parameter and (c) Z-density of MG beads, in the CG simulations with GN_DOPS. The corresponding time evolution for the GN_PCPS model can be seen in (d), (e) and (f).

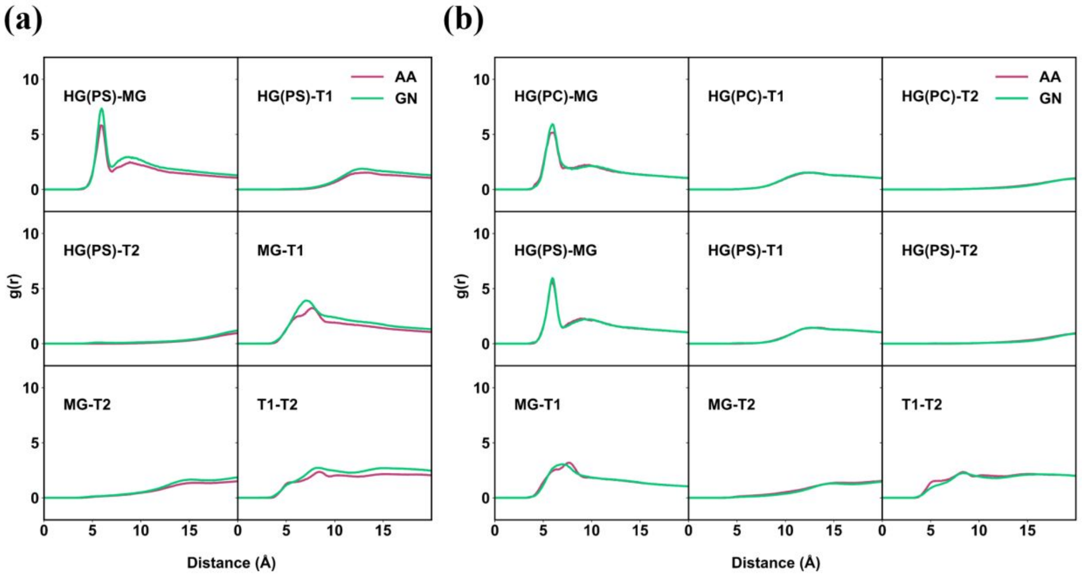

Figure S10. The RDFs between different types of beads from the mapped AA simulations, the CG simulations using the (a) GN_DOPS model and (b) GN_PCPS model.

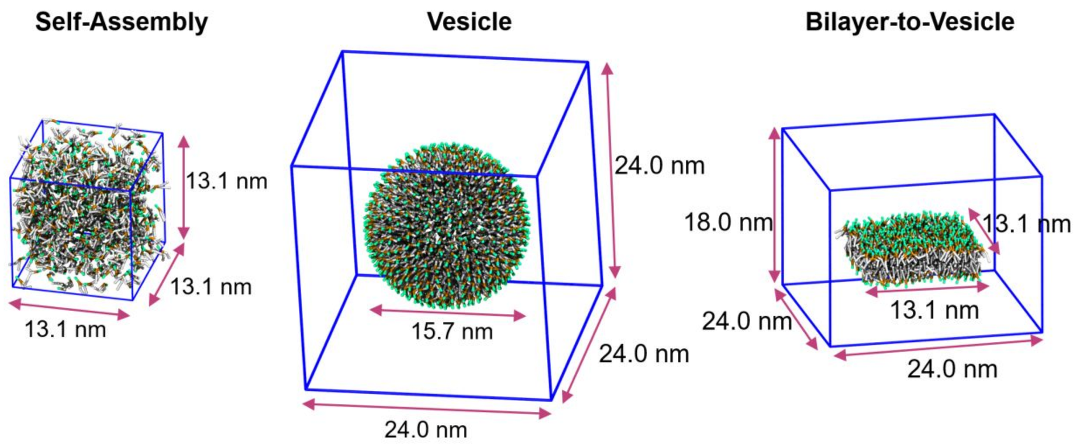

Figure S11. The initial configurations of lipids in the (a) self-assembly, (b) vesicle and (c) bilayer-to-vesicle simulations.

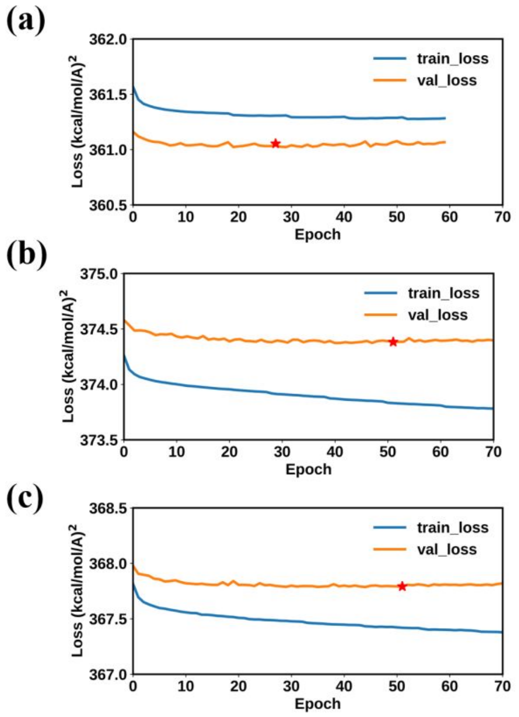

Figure S12. Training and validation loss as a function of the training epoch for the (a) GN_DOPC_BC, (b) GN_DOPS_BC and (c) GN_PCPS_BC models. The selected model is marked with a red star.

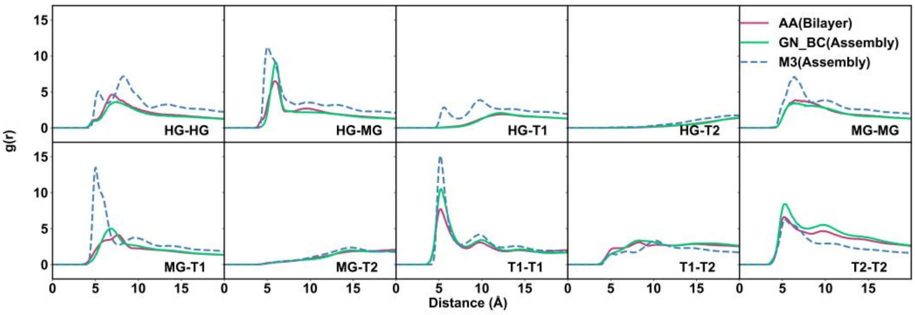

Figure S13. The RDFs between beads from the mapped AA simulations of lipid bilayer, the self-assembly simulations with GN_DOPC_BC, and the self-assembly simulations with Martini 3.

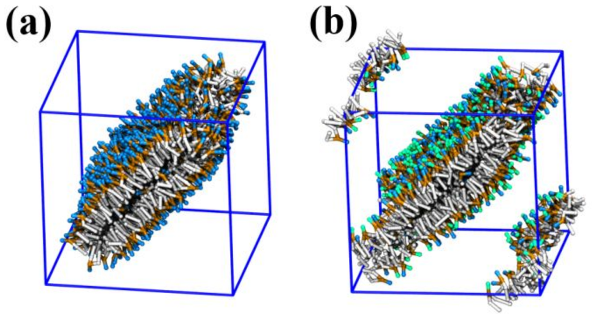

Figure S14. Snapshots from the self-assembly simulations with the (a) GN_DOPS_BC and (b) GN_PCPS_BC model.

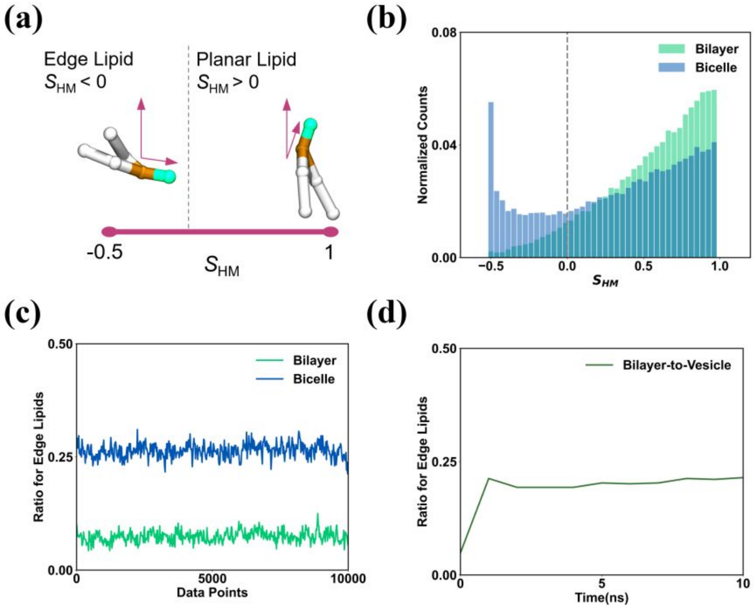

Figure S15. Lipid configurations in the training set of bicelle and bilayer. (a) The diagram illustrates how lipid configurations are distinguished by the ordering of their

head groups (S_{HM}). The edge lipids of bicelles exhibit $S_{HM} < 0$, while the planar lipids show $S_{HM} > 0$. (b) Distribution of S_{HM} and (c) the ratio of edge lipids to the total lipids in the bicelle and bilayer training set. (d) The ratio of edge lipids to the total lipids in the bilayer-to-vesicle simulation using GN_DOPC_BC.

Table S1. CG Mapping Scheme: Correspondences between all-atom, Martini, and the 6-bead CG model.

DOPC			DOPS	
AA	Martini	GN_DOPC	AA	GN_DOPS
Atom Index	Label[a]	Label	Atom Index	Label
1-24	NC3, PO4	HG (PC)	1-17	HG (PS)
25-44	GL1, GL2	MG	18-37	MG
45-66	C1A, D2A	T1	38-59	T1
67-91	C3A, C4A	T2	60-84	T2
92-113	C1B, D2B	T1	85-106	T1
114-138	C3B, C4B	T2	107-131	T2

[a]The topology files for these beads are available on the Martini website (cgmartini.nl).

Table S2. Hyperparameter search for GNN-based CG lipid force fields training. The values selected for the final models are bolded.

Hyperparameter	Value tested
Number of interaction layers	[3, **4**]
Number of RBF	[10, **18**, 64, 128]
Lower cutoff (Å)	[0, 1, **3**]
Upper cutoff (Å)	[12, **15**, 20]

Table S3. The wall-clock time of simulations using AA, Martini and GN_DOPC models. *MSD*(2) is defined as the MSD value at a time interval of 2 ns. The speed-up factor represents the relative speed-up between the CG models and the AA model, calculated based on the wall-clock time and *MSD*(2).

	Wall-clock Time (hours/ns)	*MSD*(2) (nm^{-2})	Speed-up Factor[a]
AA	0.42	0.47	1
Martini	0.007	1.11	141.6
GN_DOPC	0.56	5.90	9.4

[a] $$Speed-up\ Factor = \frac{\left(\dfrac{MSD(2)}{\text{Wallclock Time}}\right)_{\text{Model}}}{\left(\dfrac{MSD(2)}{\text{Wallclock Time}}\right)_{\text{AA}}}$$
